# Atomic layer deposition for efficient oxygen evolution reaction at Pt/Ir catalyst layers

**DOI:** 10.3762/bjnano.11.79

**Published:** 2020-06-22

**Authors:** Stefanie Schlicht, Korcan Percin, Stefanie Kriescher, André Hofer, Claudia Weidlich, Matthias Wessling, Julien Bachmann

**Affiliations:** 1 Friedrich-Alexander-Universität Erlangen-Nürnberg, Chair ’Chemistry of Thin Film Materials’, IZNF, Cauerstr. 3, 91058 Erlangen, Germany; 2 DWI-Leibniz Institute for Interactive Materials, Forckenbeckstr. 50, 52074 Aachen, Germany; 3 RWTH Aachen University Aachener Verfahrenstechnik-Chemical Process Engineering, Forckenbeckstr. 51, 52074 Aachen, Germany; 4 DECHEMA-Forschungsinstitut, Theodor-Heuss-Allee 25, 60486 Frankfurt am Main, Germany; 5 Saint Petersburg State University, Institute of Chemistry, Universitetskii pr. 26, 198504 St. Petersburg, Russia

**Keywords:** atomic layer deposition (ALD), oxygen evolution reaction (OER), redox flow battery, vanadium–air redox flow battery (VARFB)

## Abstract

We provide a direct comparison of two distinct methods of Ti felt surface treatment and Pt/Ir electrocatalyst deposition for the positive electrode of regenerative fuel cells and vanadium–air redox flow batteries. Each method is well documented in the literature, and this paper provides a direct comparison under identical experimental conditions of electrochemical measurements and in identical units. In the first method, based on classical engineering, the bimetallic catalyst is deposited by dip-coating in a precursor solution of the salts followed by their thermal decomposition. In the alternative method, more academic in nature, atomic layer deposition (ALD) is applied to the felts after anodization. ALD allows for a controlled coating with ultralow noble-metal loadings in narrow pores. In acidic electrolyte, the ALD approach yields improved mass activity (557 A·g^−1^ as compared to 80 A·g^−1^ at 0.39 V overpotential) on the basis of the noble-metal loading, as well as improved stability.

## Introduction

Reversible electrochemical energy storage devices such as rechargeable batteries, redox flow batteries (RFBs) and regenerative fuel cells (bifunctional devices able to work as electrolyzers and fuel cells) are at the forefront of a renewable energy economy as they allow one to overcome the intermittency of renewable energy sources such as solar and wind power [1–3]. The water oxidation (oxygen evolution reaction, OER) and its reverse, the oxygen reduction reaction (ORR) represent the limiting half-reaction of regenerative fuel cells [4–5], of some batteries (metal–air batteries) [6–7] and of some RFBs (such as the vanadium–air RFB). The positive electrode of these devices has to perform the challenging OER and ORR on one material system. A bimetallic Pt/Ir electrocatalyst is used most commonly as it provides minimal overpotentials under strongly acidic conditions [3,8–10]. The significant cost of theses noble metals renders it necessary to minimize their loading and maintain optimal access of the electrolyte to every active site of their surface. Numerous studies have been dedicated to the development of such bimetallic catalysts in various compositions [11], using various coating methods [12] as well as particles of various sizes [13] and shapes [14]. However, each study is presented as a self-sufficient piece of work with limited critical comparison to the state of the art. The conditions under which electrochemical performance is quantified (electrode substrate, electrolyte composition and pH value, temperature, or reference electrode), or even the choice of performance parameters (current density at a given overpotential, mass activity based on noble-metal loading, or overpotential for a given current), are usually different. Hence, often direct comparisons between catalysts of different types are not possible.

The goal of the present paper is to provide such a direct comparison between two very distinct preparation methods of bimetallic Pt/Ir electrocatalysts. For both catalyst types, we will consider one electrode substrate (titanium felt) [15], one electrolyte (0.5 M H_2_SO_4_ at room temperature), one electrochemical characterization (linear voltammetry performed at room temperature), and we will quantify (a) current densities, (b) mass activites (given a certain noble-metal loading) and (c) stability (quantified as a change in current density after bulk electrolysis).

We consider here two distinct catalyst preparation methods. As a standard method used in the engineering context, we perform an acid etch of the titanium fibers (to generate surface roughness and thereby increase the specific surface area), followed by dip-coating of a noble metal salt precursor solution on the Ti support and subsequent thermal decomposition to the corresponding elements [16–18]. As an academic method yielding better control of the electrode surface geometry, we perform an “anodization” of the Ti fibers to generate an ordered porous layer, followed by atomic layer deposition (ALD) of the Pt/Ir catalyst [19–20].

In the former procedure, which hereafter we will refer to as the “thermal decomposition” method, we aimed for a coating of salts of 2.0 mg·cm^−2^, corresponding to 750 μg·cm^−2^ of noble metal. Using the ALD method (the latter one), the loadings achieved were between 50 and 70 μg·cm^−2^. In both cases, the composition achieved is near the optimal 1:1 ratio of Pt to Ir. The electrodes based on the thermal decomposition method have been used and characterized in the past [6,21], and the material from the ALD method has been investigated extensively in [22–23]. In particular, we have determined particle sizes by transmission electron microscopy (TEM), investigated the homogeneously mixed nature of the Pt/Ir catalyst by X-ray diffraction (XRD), selected-area electron diffraction (SAED), and X-ray photoelectron spectroscopy (XPS). We have also examined various ALD mixing ratios and mixing procedures. Since the material characterization has been performed to some level of detail for both systems in previous publications, we will in this paper only repeat simple characterization of each material system for the sake of comparison between them, and we will focus on the performance comparison.

## Results and Discussion

### Catalyst coating: thermal decomposition

In the thermal decomposition method, platinum and iridium metal salts (the perchlorometallates) were dissolved in 1:1 ratio in butanol to form the catalyst precursor solution. The Ti felts (two distinct porosities, which in the following we will call A and B) were pretreated in acid (12 M HCl at 85 °C) in order to generate surface roughness, then dip-coated with the catalyst precursor solution and dried. The dip-coating was repeated until the desired loading was achieved as determined by gravimetric methods. Finally, the samples were calcinated at 450 °C to generate the metallic catalyst.

**Figure 1:**
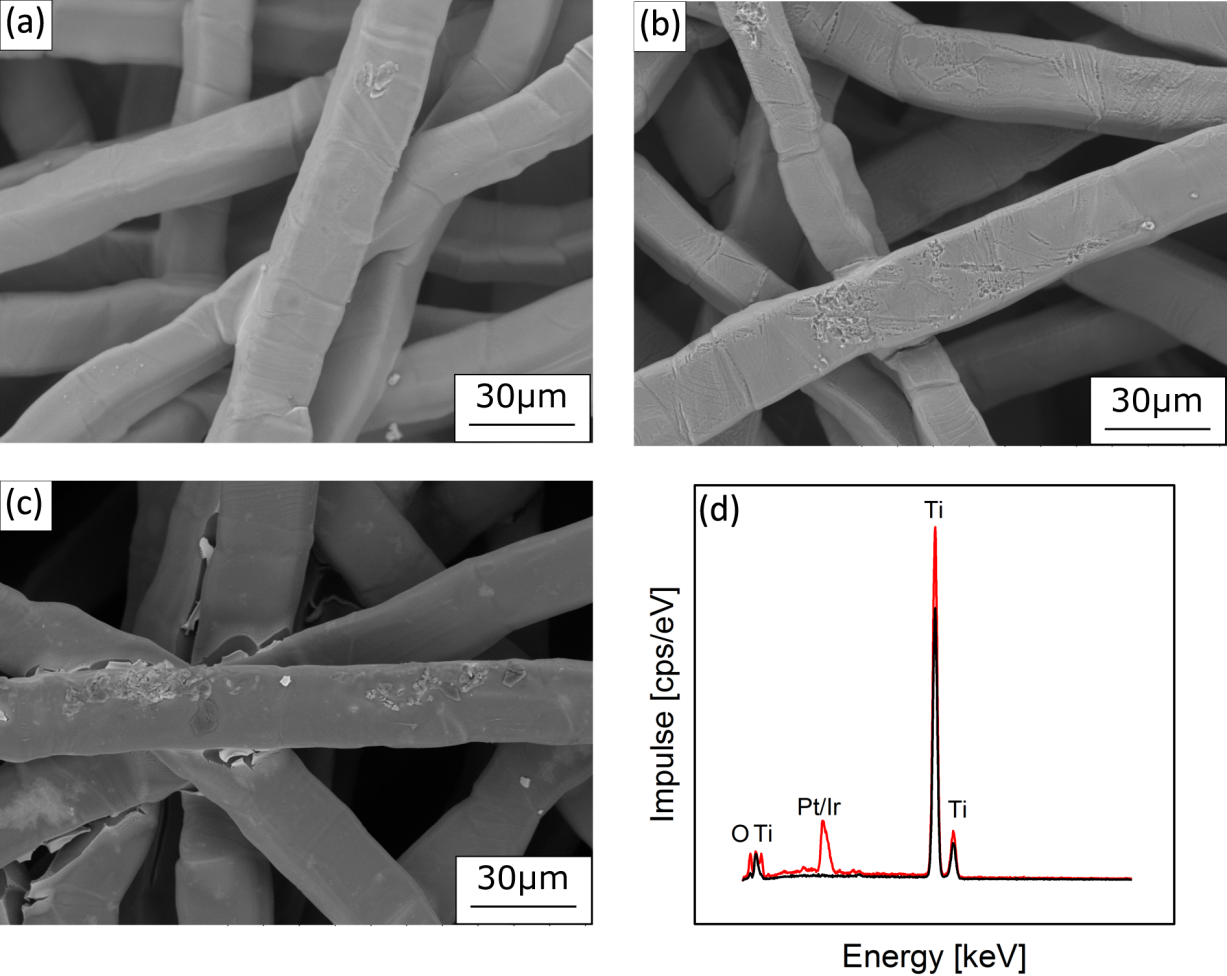
Scanning electron micrographs of (a) a commercial titanium felt, (b) the acid-treated felt, (c) the electrode after catalyst coating using the thermal decomposition method. (d) Energy-dispersive X-ray analysis of the titanium felt (black line) and the catalyst-coated electrode (red line).

Figure 1 presents the SEM and EDX characterization of the titanium felts obtained by this method. Figure 1a presents the untreated felt, the titanium fibers of which are clearly visible. Figure 1b shows the felt after acid etching. The surface roughness has increased, thereby increasing the specific surface area. The acid treatment also generates a homogeneously thin and stable TiO_2_ layer, which provides corrosion resistance while maintaining low transfer coefficients [24].

After coating with the catalyst layer (Figure 1c), a rather thick layer of the order of 1 μm is observed. Some cracks can be detected on the egdes of the coating where the fibers cross, due to low transport rates at these locations and high mechanical stress. The existence of the noble metals is proven by energy-dispersive X-ray analysis (EDX, Figure 1d), although the Pt and Ir lines overlap too much for distinct quantification by EDX. Apart from the prominent Ti peak, the presence of an oxide layer is demonstrated by a significant O signal.

**Figure 2:**
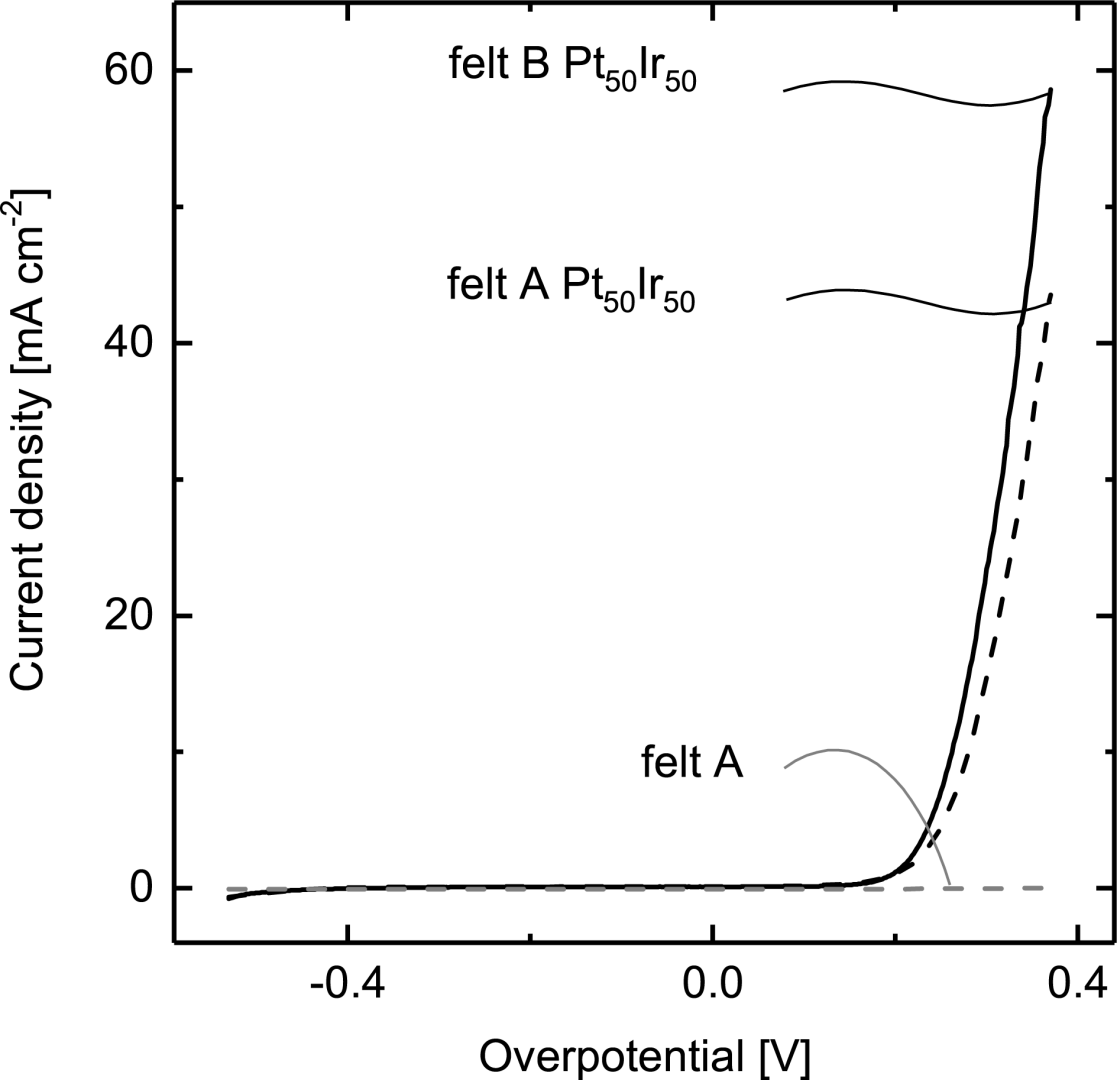
Linear sweep voltammograms for Ti felts A and B coated with catalyst (1:1 Pt/Ir 0.75 mg·cm^−2^) by the thermal decomposition method, as compared to non-coated felt A.

Figure 2 presents the linear sweep voltammograms obtained in the OER region for catalyst-coated samples (felts A and B) as compared to their non-coated Ti felt references. On the current scale shown here, the exponential current increase starts at an overpotential (η) of about 0.2 V and reaches nearly 60 mA·cm^−2^ at η = 0.4 V on felt B, while felt A (the less porous version) is limited to 45 mA·cm^−2^. Alternatively, felt B can be described as reducing the overpotential by approximately 30 mV. Given our loadings (0.75 mg·cm^−2^), the current density (on felt B) corresponds to a mass activity of 80 A·g^−1^ on a noble metal basis.

### Catalyst coating: atomic layer deposition

Atomic layer deposition (ALD) is an attractive thin film technique that allows for a conformal coating of not only planar substrates but also of highly porous ones [12,19–20,25]. This method is based on well-defined self-limiting surface reactions combined to deposit thin layers with highly uniform thickness. At least two precursors chemisorb with the active sites on the substrate surface in an alternating manner, in order to deposit the solid atom layer by atom layer. Thus, ALD provides a precise control over the film thickness at the angstrom level. Additionally, the composition of the film can be varied systematically by repeating and combining individual ALD cycles of two or more materials. The deposition of platinum and iridium as a binary catalyst can be realized at very low loadings in the range of micrograms per square centimeter [26]. Specifically, the applicability of ALD regarding Ir and bimetallic Pt/Ir catalysts, the homogeneous mixing of both elements in small nanoparticles, the coating of deep pores with them, and the adequacy of anodization with ALD as a surface treatment of Ti felts have all been demonstrated in the recent past [22–23].

**Figure 3:**
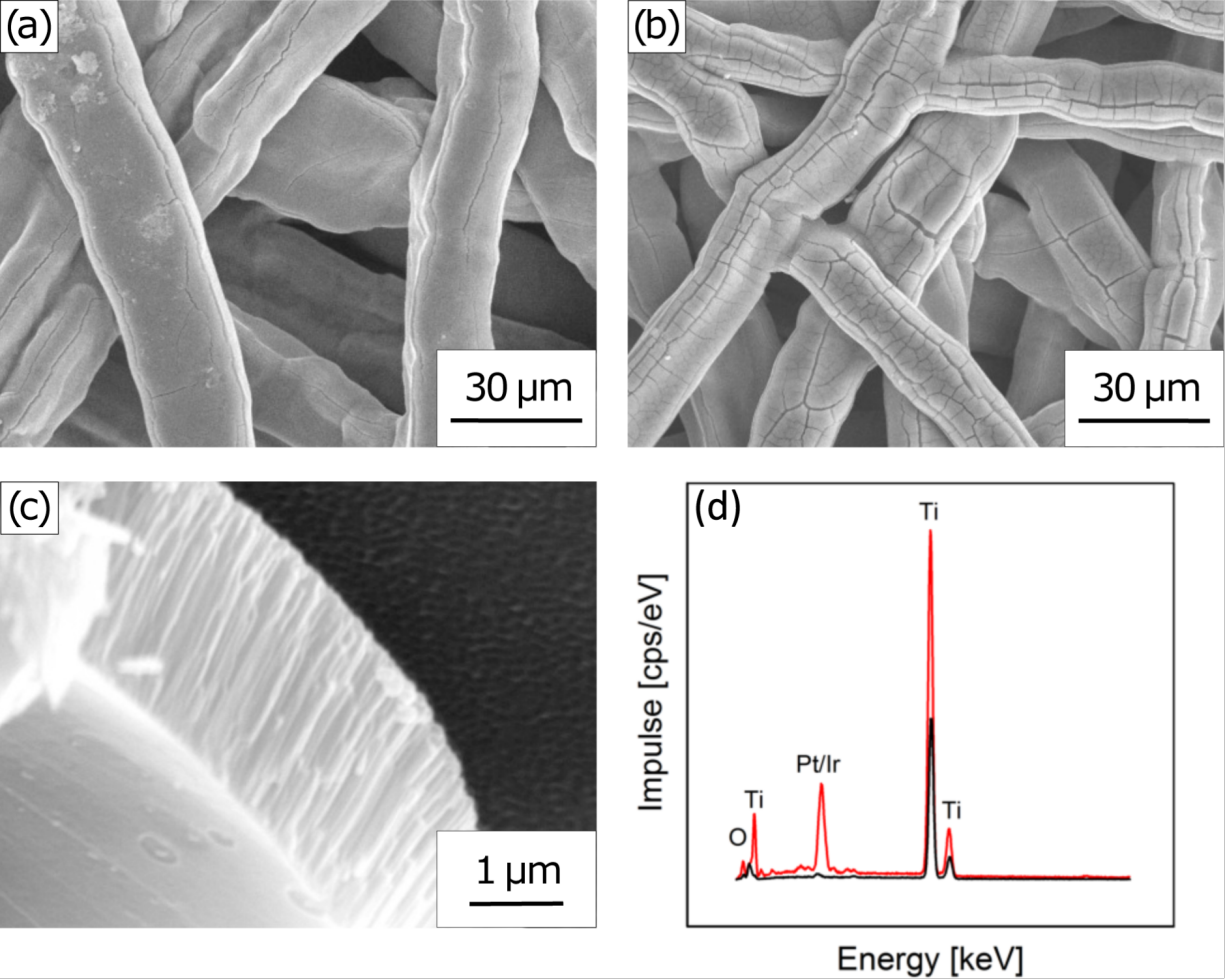
(a–c) Scanning electron micrographs of titanium felts with different porosities after anodization in 0.5 wt % NH_4_F in glycerol: (a,c) type A at two magnification levels, and (b) type B. (d) Energy-dispersive X-ray analysis evidences the presence of platinum and iridium after atomic layer deposition.

Anodization is performed by applying a large positive voltage (+40 V) to the felt in a NH_4_F/glycerol solution for 2 h and results in the formation of a ca. 2.5 μm thick oxide layer consisting of parallel nanometer-wide pores (Figure 3). This layer offers an increase in specific surface area as compared with the smooth fibers of the commercial felt, with the specific parameters used here by a factor of approximately 70. The small diameter of the pores, however, means that they are not accessible to all coating methods. In particular, we have observed that thermal decomposition coating of anodized felts yields electrodes of poor electrocatalytic performance.

In contrast to this, a Pt/Ir mixture can be deposited along the TiO_2_ tube walls by atomic layer deposition (ALD). Energy-dispersive X-ray analysis confirms the presence of all elements expected (Figure 3d). The metallic nature of the ALD deposits, the lack of any segregation between Pt and Ir, and the metal oxide nature of the surfaces have also been demonstrated in recent publications by X-ray diffraction, selected-area electron diffraction and transmission electron microscopy [22–23]. Herein, we investigate two different catalyst loadings: (1) Ir_ALD25_Pt_ALD10_, i.e., 25 ALD cycles Ir followed by 10 ALD cycles Pt, yielding a predominantly Pt-covered surface and (2) (Ir_ALD5_Pt_ALD5_)_2_, i.e., 5 ALD cycles Ir + 5 ALD cycles Pt repeated twice, yielding a more homogeneous mixture within each particle. The thickness of the catalyst layers is approximately two nanometers depending on the Ir/Pt mixture. The exact loadings were quantified with inductively coupled plasma optical emission spectrometry (ICP-OES) and found to be in the range of 48–66 μg·cm^−2^ (Table 1).

**Table 1:** Noble-metal loadings of the four samples presented in Figure 4, determined by ICP-OES analysis.

felt type	ALD parameters	Pt [μg·cm^−2^]	Ir [μg·cm^−2^]	Pt + Ir total [μg·cm^−2^]

A	Ir_ALD25_Pt_ALD10_	17	49	66
A	(Ir_ALD5_Pt_ALD5_)_2_	12	42	54
B	Ir_ALD25_Pt_ALD10_	17	31	48
B	(Ir_ALD5_Pt_ALD5_)_2_	28	20	48

**Figure 4:**
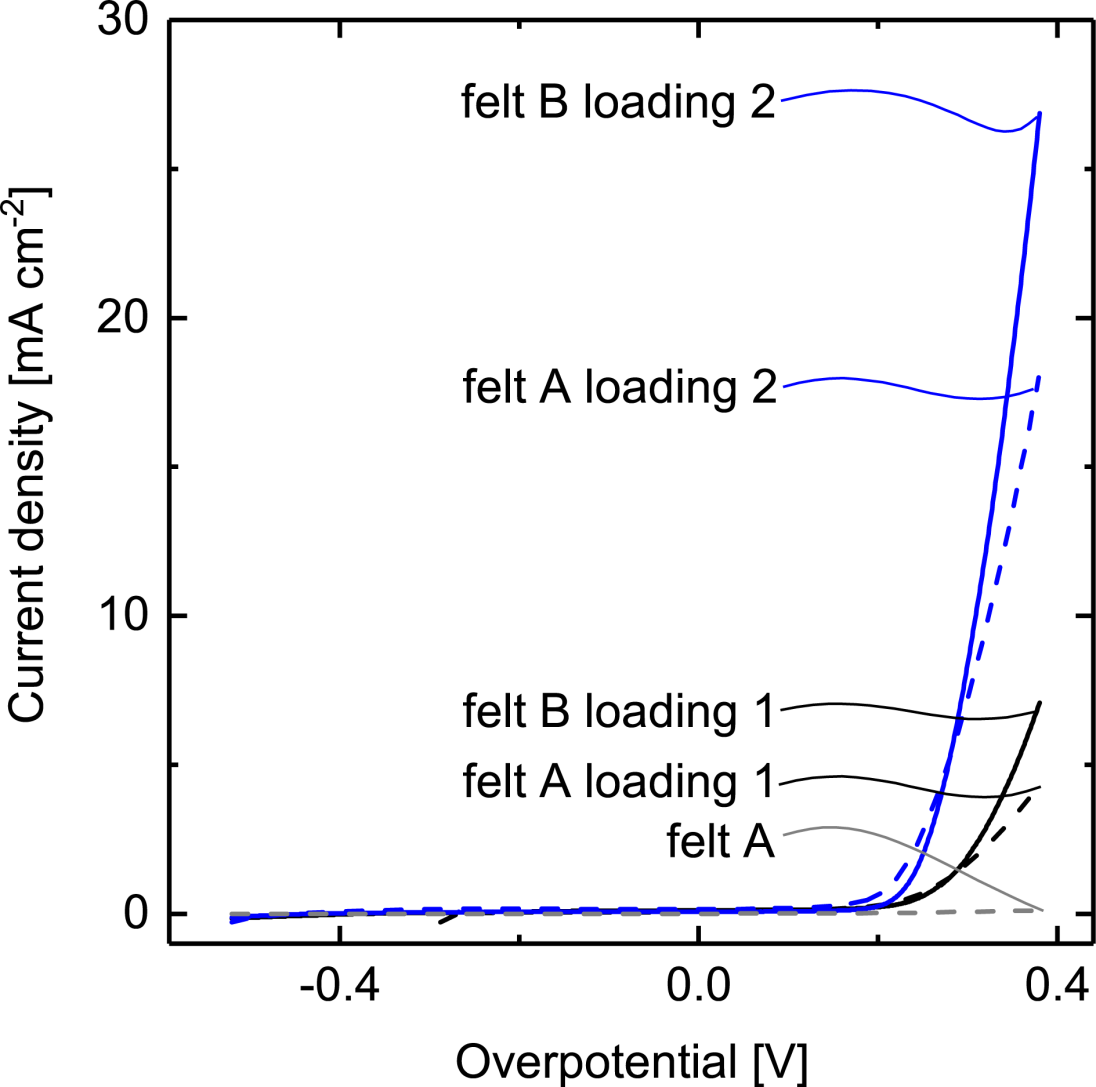
Linear sweep voltammograms of titanium felts with two different porosities, felt A (dashed line) and felt B (solid line), and two different catalyst loadings Ir_ALD25_Pt_ALD10_ (black) and (Ir_ALD5_Pt_ALD5_)_2_ (blue). Table 1 lists the noble-metal loadings of the corresponding four samples determined by ICP-OES.

The linear sweep voltammograms presented in Figure 4 and recorded under the exact same conditions as the thermal decomposition samples (Figure 2) are qualitatively similar to them. Quantitatively, however, their performance depends on both the felt type and the catalyst loading. The better mixed catalyst (Ir_ALD5_Pt_ALD5_)_2_ yields OER current densities four times larger than Ir_ALD25_Pt_ALD10_ for both types of titanium felt. This is most likely related to the more prominent presence of catalytically more active Ir at the surface. The geometric effect of felt porosity is also prevalent on the graphs. The best sample achieves 26.8 μA·cm^−2^ at η = 0.4 V, which corresponds to a mass activity of 557 A·g^−1^. This catalyst coating yields (as compared to the thermal decomposition method) a somewhat lower current density for a very much lower noble metal coating. In other words it shows an activity per gram of noble metal improved by a factor of seven in comparison to the electrodes prepared with the thermal decomposition method. Of course, this result does not imply an inherently different chemical activity. It simply means that the combination of a large specific surface area generated by anodization with a controlled deposition inside the nanoscale pores presents the catalytically active surface in a particularly advantageous manner.

### Catalyst stability

**Figure 5:**
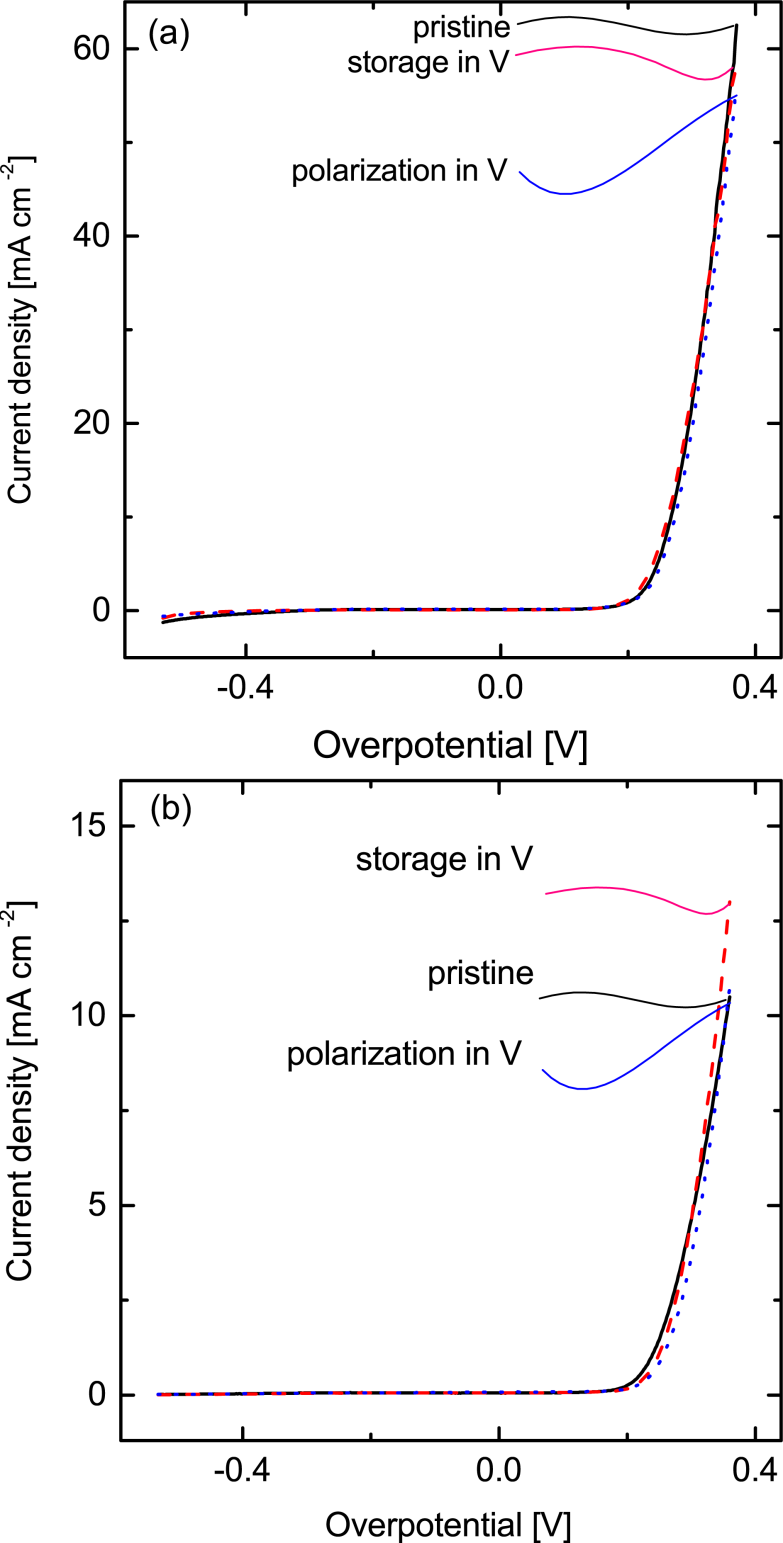
Stability analysis of catalyst coated titanium felts using a) the thermal deposition method and b) the ALD method. Pristine (black solid line) storage in V (red dashed line) and polarization in V (blue dotted line).

The combination of Ti as the electrode substrate with Pt and Ir as catalyst(s) is one of the few that ensure stability against the strongly acidic and oxidizing conditions of a reversible fuel cell. However, OER/ORR electrodes in some other electrochemical energy storage devices may have to endure even more harsh conditions. For example, in vanadium–air redox flow batteries the crossover of vanadium ions through the ion exchange membrane renders the electrolyte even more corrosive. To simulate electrode ageing under accelerated conditions, we study the activity loss of catalyst-coated electrodes after soaking for one day in a 1.6 M vanadium electrolyte (in H_2_SO_4_), followed by polarization to values between 0.3 and 1.6 V (five voltammetric cycles, 5 mV·s^−1^, approximately 43 min). Figure 5a shows that the samples obtained with the thermal decomposition method suffer a loss of activity upon storage, followed by a further subsequent loss upon polarization (8% overall). Their ALD counterparts, however, remain unaffected (Figure 5b). Thus, a Pt/Ir catalyst coated with the ALD method on anodized felts results in a significant stability gain with respect to the traditional thermal decomposition method of catalyst coating.

## Conclusion

We have demonstrated and compared two different methods for preparing a bimetallic catalyst electrode for application in electrochemical energy storage and release in electrolyzer/fuel cells, in vanadium–air redox flow batteries, or in other related devices. The preparation bases on the coating of commercial titanium felts, the surface area of which is enhanced by either thermal acid etching or electrochemical “anodization”. Platinum and iridium catalysts are deposited either by dip-coating/thermal decomposition from a solution of noble metal salts or by atomic layer deposition. The ALD method enables one to optimize the activity for the oxygen evolution reaction using low noble-metal loadings (48–66 μg·cm^−2^). In comparison to the electrodes prepared by thermal decomposition, the activity per gram of noble metal is increased by about 600% (557 A·g^−1^ at η = 0.39 V). Whether or not this activity gain remains as large in the membrane-electrode assemblies (MEAs) of full devices depends on the accessibility of the nanostructured surface to the polyelectrolyte. Furthermore, for application to regenerative fuel cells the studies will have to be repeated for the ORR and expanded to stability measurements on very long operation timescales (years) or accelerated degradation tests. Under the operating conditions of a vanadium–air redox flow battery, however, the ALD-treated electrodes show a significant stability improvement towards the strongly acidic vanadium electrolyte.

## Experimental

Titanium felt A (ST/Ti/20/1200/70, Bekaert, Belgium) and titanium felt B (ST/Ti/20/350/50, Bekaert, Belgium) of 30 mm × 10 mm were used as support materials for the catalyst coatings.

### Catalyst coating by the thermal decomposition method

The support materials were pretreated in a 12 M HCl (Alfa-Aesar, Germany) solution at 85 °C for 3 min. Subsequently, they were rinsed with H_2_O and stored in a vacuum oven at 50 °C overnight. The coating solution for the thermal decomposition method consists of Pt_50_Ir_50_. The platinum salt (chloroplatinic acid hexahydrate, H_2_PtCl_6_·6H_2_O, Sigma-Aldrich, Germany) and iridium salt (hydrogen hexachloroiridate(IV) hydrate, H_2_IrCl_6_·*x*H_2_O, Sigma-Aldrich, Germany) were dissolved in 1-butanol (*>*99.5%, Carl Roth, Germany). The resulting 3 wt % precursor solutions were mixed 1:1 to produce the Pt_50_Ir_50_ coating solution. The targeted loading for the catalyst was 2 mg·cm^−2^ of salt based on macroscopic sample area. Only 1 cm^2^ of the support material was dipped into the well-mixed coating solution for 3 min. Subsequently, the sample was placed in an oven at 90 °C for 20 min. Based on the weight gain these steps were repeated until the desired loading was reached. Subsequently, the sample was calcinated in the oven at 450 °C for 60 min under synthetic air.

### Catalyst coating by the atomic layer deposition method

The preparation broadly follows the procedure described in detail in [22]. In short, the titanium felts were anodized in a solution of 0.5 wt % NH_4_F in glycerol at +40 V for two hours using two graphite plate counter electrodes. Subsequently, they were rinsed with water and ethanol several times. Catalyst coating was performed by atomic layer deposition in a commercial Gemstar 6 reactor from Arradiance. Platinum and iridium were deposited using ethylcyclopentadienyl-1,3-cyclohexadiene-iridium(I) ((EtCp)Ir(CHD)), trimethyl(methylcyclopentadienyl)platinum (MeCpPtMe_3_) and ozone as precursors. Ozone was produced from dioxygen in an ozone generator model BMT 803N. The deposition was carried out at 220 °C and the iridium and platinum precursor bottles were maintained at 90 and 50 °C, respectively. The durations for pulsing, exposure and pumping were set to 500 ms, 30 s and 90 s, while for the metal precursors the pulsing/exposure time was repeated two or four times within one ALD cycle. Two different loading cycles were performed: (1) 25 cycles Ir + 10 cycles Pt, named as Ir_ALD25_Pt_ALD10_ (2) 5 cycles Ir + 5 cycles Pt + 5 cycles Ir + 5 cycles Pt, named as (Ir_ALD5_Pt_ALD5_)_2_.

### Analyses

The morphology of the titanium felts was investigated using either a scanning electron microscope JEOL JSM 6400 equipped with an EDX detector from SAMx or a Hitachi table-top TN3030+. The loading of the Pt/Ir catalyst was determined by inductively coupled plasma optical emission spectrometry (ICP-OES, Optima 8300, Perkin Elmer). For this analysis, four-point calibrations (50, 10, 1 and 0.1 ppm) were performed by diluting certified standards. The samples were measured in triplicate and mean values are reported.

### Electrochemical setup

For electrochemical investigations, a standard three-electrode setup was used. The cell was filled with 100 mL of 0.5 M H_2_SO_4_ (AVS TITRINORM®, VWR) as the electrolyte. A Hg/Hg_2_SO_4_ reference electrode filled with 0.5 M H_2_SO_4_ (Sensortechnik Meinsberg) mounted in a Haber–Luggin capillary or a Ag/AgCl reference electrode was used. A platinum (coated titanium mesh) electrode was used as the counter electrode (Magneto Special Anodes B.V., The Netherlands). The working electrodes consist of the prepared catalyst-coated titanium electrodes. Electrochemical characterization was carried out with a potentiostat/galvanostat (PGSTAT302N, Metrohm GmbH and Gamry Interface 1000) at room temperature.

### Electrochemical measurements

Prior to the electrochemical experiments, the working electrodes were activated through the following steps. Firstly, cyclic voltammograms (ten cycles) were recorded starting at the open-circuit potential (OCP) and oscillating between +0.3 V and +1.6 V vs NHE. Secondly, chronoamperometry was carried out for 15 min at 1.24 V vs NHE. After activation, the electrolyte (0.5 M H_2_SO_4_) was deaerated by bubbling inert gas for 20 min. During the subsequent measurements, an inert gas flow was maintained over the surface of the liquid electrolyte. Following this pretreatment, the electrochemical analyses consisted of linear sweep voltammograms (LSV) recorded from 0.7 to 1.6 V vs NHE (three scans, 1 mV·s^−1^). Before every LSV scan a chronoamperometry was performed at the initial potential of the subsequent LSV scan for 2 min. To investigate the catalyst stability, the sample was stored overnight in 1.6 M vanadium electrolyte solution (GfE GmbH, Germany). Thereafter, the electrochemical analyses were repeated (in H_2_SO_4_). Subsequently, polarisation in GfE electrolyte solution was carried out without gas supply to the cell by performing five voltammetric cycles from OCP between 0.3 and 1.6 V vs NHE (five cycles, 5 mV·s^−1^, stop potential OCP). This corresponds to approximately 43 min under various potentials in the vanadium electrolyte. Subsequently, the electrochemical analyses were repeated (in pure sulfuric acid electrolyte). In all cases the current density is defined as the current per unit of macroscopic sample area. The first voltammogram was not taken into account, the subsequent three measurements were averaged for reporting.

## References

[1] Yan D, Li Y, Huo J, Chen R, Dai L, Wang S (2017). Adv Mater (Weinheim, Ger).

[2] Lee Y, Suntivich J, May K J, Perry E E, Shao-Horn Y (2012). J Phys Chem Lett.

[3] Jiao Y, Zheng Y, Jaroniec M, Qiao S Z (2015). Chem Soc Rev.

[4] Sadhasivam T, Dhanabalan K, Roh S-H, Kim T-H, Park K-W, Jung S, Kurkuri M D, Jung H-Y (2017). Int J Hydrogen Energy.

[5] Carmo M, Fritz D L, Mergel J, Stolten D (2013). Int J Hydrogen Energy.

[6] Hosseiny S S, Saakes M, Wessling M (2011). Electrochem Commun.

[7] Zhu Y G, Jia C, Yang J, Pan F, Huang Q, Wang Q (2015). Chem Commun.

[8] Jörissen L (2006). J Power Sources.

[9] Dresp S, Luo F, Schmack R, Kühl S, Gliech M, Strasser P (2016). Energy Environ Sci.

[10] Zhang J, Zhao Z, Xia Z, Dai L (2015). Nat Nanotechnol.

[11] Cheng Y, Jiang S P (2015). Prog Nat Sci: Mater Int.

[12] Schlicht S, Haschke S, Mikhailovskii V, Manshina A, Bachmann J (2018). ChemElectroChem.

[13] Jirkovský J, Makarova M, Krtil P (2006). Electrochem Commun.

[14] Tung C-W, Hsu Y-Y, Shen Y-P, Zheng Y, Chan T-S, Sheu H-S, Cheng Y-C, Chen H M (2015). Nat Commun.

[15] Shin S, Choi Y-W, Choi J (2013). Mater Lett.

[16] Song S, Zhang H, Ma X, Shao Z, Baker R T, Yi B (2008). Int J Hydrogen Energy.

[17] Honoré Kondro K, Ouattara L, Trokourey A, Bokra Y (2008). Bull Chem Soc Ethiop.

[18] Li F-M, Gao X-Q, Li S-N, Chen Y, Lee J-M (2015). NPG Asia Mater.

[19] Puurunen R L (2005). J Appl Phys.

[20] Bachmann J (2014). Beilstein J Nanotechnol.

[21] Kriescher S M A, Kugler K, Hosseiny S S, Gendel Y, Wessling M (2015). Electrochem Commun.

[22] Schlicht S, Barr M K S, Wu M, Hoppe P, Spiecker E, Peukert W, Bachmann J (2018). ChemElectroChem.

[23] Schlicht S, Büttner P, Bachmann J (2019). ACS Appl Energy Mater.

[24] Devilliers D, Dinh M T, Mahé E, Krulic D, Larabi N, Fatouros N (2006). J New Mater Electrochem Syst.

[25] Zazpe R, Knaut M, Sopha H, Hromadko L, Albert M, Prikryl J, Gärtnerová V, Bartha J W, Macak J M (2016). Langmuir.

[26] King J S, Wittstock A, Biener J, Kucheyev S O, Wang Y M, Baumann T F, Giri S K, Hamza A V, Baeumer M, Bent S F (2008). Nano Lett.

